# Editorial: Molecular biology of plant-fungal interactions

**DOI:** 10.3389/fpls.2024.1392149

**Published:** 2024-03-05

**Authors:** Dayong Li, Fengming Song, Tesfaye Mengiste

**Affiliations:** ^1^Ministry of Agriculture and Rural Affairs Key Lab of Molecular Biology of Crop Pathogens and Insects, Institute of Biotechnology, Zhejiang University, Hangzhou, China; ^2^Key Laboratory of Biology of Crop Pathogens and Insects of Zhejiang Province, Institute of Biotechnology, Zhejiang University, Hangzhou, China; ^3^State Key Laboratory for Rice Biology and Breeding, Institute of Biotechnology, Zhejiang University, Hangzhou, China; ^4^Department of Botany and Plant Pathology, Purdue University, West Lafayette, IN, United States

**Keywords:** plant-fungal interactions, fungal virulence, host defense mechanisms, genetic engineering, elicitors, effectors, disease resistance

Plant fungal pathogens represent a highly diverse and formidable group of biotic stressors that significantly impact agricultural productivity and economic outcomes. The complexity of plant-fungal interactions arises from the intricate processes involving both the plant and the fungal organisms. To combat fungal pathogens, plants have evolved a sophisticated two-tiered immune receptor system designed to recognize and mount responses against such pathogens (Ngou et al., 2022). Conversely, fungal pathogens have developed mechanisms to evade and suppress plant defenses, creating a dynamic interplay of resistance and susceptibility (Oliveira-Garcia and Valent, 2015). In light of these challenges, there is a pressing need for innovative control strategies. A deeper understanding of the adaptive strategies employed by fungal pathogens and the mechanisms through which plants modulate their resistance or susceptibility during these interactions is crucial. Such knowledge will be instrumental in advancing the development of effective disease control methods in plants, thereby safeguarding agricultural production and economic viability.

To enhance discussion in this vibrant area of study, we have introduced the Research Topic entitled “*Plant-Fungal Interactions*.” This Research Topic is enriched with contributions from eminent researchers, encompassing both innovative primary research articles and insightful review papers dedicated to exploring the nuances of plant-fungal relationships. With a collection of five original research papers and two thorough reviews, this series aims to spotlight the forefront of research and progress in the domain of plant and fungal biology, emphasizing the complex interactions between these two biological kingdoms. The articles within this Research Topic rigorously investigate molecular mechanisms, defense strategies, and their implications for improving crop resilience. Collectively, these contributions significantly advance our understanding of these intricate biological interactions, marking crucial progress in the disciplines of plant pathology and agricultural biotechnology. An overview of the content is summarized below.

Recent investigations by Nie et al. presented a detailed examination of MoVcpo, a novel protein from the rice pathogen *Magnaporthe oryzae*, highlighting its dual role in the fungal pathogen’s virulence and the activation of rice’s defense mechanisms. It demonstrates how MoVcpo triggers rice defense responses, including the induction of reactive oxygen species and upregulation of defense-related genes, enhancing rice resistance to the pathogen. The study underscores MoVcpo’s potential as a target for developing rice varieties with improved resistance to rice blast disease. This research provides significant insights into the molecular interactions between rice and *M. oryzae*, offering new avenues for crop protection strategies.

In a groundbreaking study, Zhu et al. explored BcCDI1, a protein from *Botrytis cinerea*, highlighting its dual role in inducing plant cell death and eliciting immune responses in plants like tobacco. Despite its significant role in plant defense, BcCDI1 does not alter the course of *B. cinerea* infection, indicating a complex interaction between pathogen virulence and host defense mechanisms. The research underscores the importance of understanding such proteins for developing disease-resistant crops and provides insights into the molecular dynamics of plant-pathogen interactions.

Utilizing advanced transcriptome analysis, Zhao et al. identified and characterized long non-coding RNAs in Kentucky bluegrass responding to rust infection, revealing their significant role in plant defense. The study utilized full-length transcriptome sequencing to explore the response of Kentucky bluegrass to rust infection, identifying differentially expressed long non-coding RNAs (lncRNAs) and genes. It revealed 105 differentially expressed lncRNAs and 15,711 differentially expressed genes, highlighting their roles in plant hormone signal transduction and plant-pathogen interaction pathways. This research provides insights into the molecular mechanisms of rust resistance in Kentucky bluegrass, offering potential targets for breeding rust-resistant varieties.

Focusing on innovative genetic strategies, Raruang et al. successfully reduced *Aspergillus flavus* infection and aflatoxin contamination in maize by targeting the fungal *p2c* gene through host-induced gene silencing, marking a significant advancement in crop protection. Transgenic maize lines targeting the suppression of the fungal gene showed significantly lower aflatoxin levels, attributed to RNAi-based suppression of *p2c* expression, leading to reduced fungal growth and toxin production. This approach demonstrates a promising strategy for enhancing crop resistance against fungal pathogens and minimizing aflatoxin risks, contributing valuable insights into the application of genetic engineering in agriculture.

A comprehensive review by Zhu et al. meticulously explores the complex defense mechanisms of cotton against *Verticillium dahliae*, proposing a strategic framework for breeding programs aimed at mitigating Verticillium wilt. This review delineates *V. dahliae*’s intricate infection strategies, including microsclerotia formation, nutrient competition, and suppression of the host’s immune system. In response, cotton employs strategies such as structural modification of tissues, accumulation of antifungal compounds, and activation of immune signaling pathways. Emphasizing the necessity of understanding these dynamic interactions, the review lays a solid groundwork for future genetic engineering endeavors to develop *Verticillium* wilt-resistant cotton varieties.

Plants counteract pathogen threats by activating diverse immune responses, yet adept pathogens circumvent these defenses using effectors, and virulence proteins secreted during infection (Figueroa et al., 2021). These effectors, which can function within plant extracellular spaces or directly within host cells, manipulate cellular processes to suppress immunity and foster an environment favorable for pathogen growth (Zhang et al., 2022). The pivotal role of these effectors in promoting plant vulnerability and disease advancement is highlighted through detailed studies of *Phytophthora infestans* and *Puccinia striiformis* f. sp. *tritici*, demonstrating their elaborate evasion tactics against potato and wheat defenses, respectively. Research by Xiong et al. delves into how the *Phytophthora infestans* effector Pi07586 undermines potato defenses, showcasing its increased expression during infection and its nuclear localization, which aids *P. infestans* in establishing its presence. This study points out that Pi07586 inhibits crucial genes related to defense and photosynthesis, along with significantly lowering essential plant hormones such as abscisic acid, jasmonic acid, and jasmonoyl-isoleucine, but leaves salicylic acid and indole-3-acetic acid levels unaffected. Such insights into *P. infestans*’ molecular tactics for circumventing plant immune defenses suggest new avenues for enhancing crop resilience. Meanwhile, Wu et al. delve into the diverse effector repertoire of *Puccinia striiformis* f. sp. *tritici*, shedding light on the ongoing evolutionary struggle between wheat and this pathogen. Their comprehensive review addresses the effectors’ functions, localization within cells, and interactions with host plants, emphasizing the pathogen’s strategies for evading plant immunity. Highlighting the role of advanced sequencing and bioinformatics in identifying effectors, the review confronts the challenges of such research and underscores the critical need for a deeper understanding of effector functions to develop wheat varieties resistant to disease. Both studies emphasize the essential role of effectors in compromising host immune mechanisms, altering host gene expression, and facilitating pathogen colonization, highlighting the critical need for their identification and characterization through advanced genomic and bioinformatics approaches to foster the development of crops with enhanced resistance.

Overall, the articles featured in this Research Topic collectively advance our knowledge of plant-fungus interactions, shedding light on the intricate molecular dialogues that dictate the outcomes of these relationships. By exploring both the offensive strategies employed by fungi and the defensive mechanisms of plants, this Research Topic contributes to the development of knowledge that underpins innovative strategies for crop protection and improvement. The findings presented here not only enhance our understanding of the biological underpinnings of these interactions but also pave the way for applying this knowledge toward more resilient agricultural systems. We extend our deepest gratitude to all contributors, reviewers, and editors who played a pivotal role in bringing this Research Topic to fruition. Their dedication and insightful contributions have enriched our understanding of the complex interplay between plants and fungi, marking a significant step forward in the field of plant pathology and agricultural sciences.

## Author contributions

DL: Writing – original draft. FS: Writing – review & editing. TM: Writing – review & editing.

## References

[B1] Figueroa M. Ortiz D. Henningsen E. C. (2021). Tactics of host manipulation by intracellular effectors from plant pathogenic fungi. Curr. Opin. Plant Biol. 62, 102054. doi: 10.1016/j.pbi.2021.102054 33992840

[B2] Ngou B. P. M. Ding P. Jones J. D. G. (2022). Thirty years of resistance: Zig-zag through the plant immune system. Plant Cell 34, 1447–1478. doi: 10.1093/plcell/koac041 35167697 PMC9048904

[B3] Oliveira-Garcia E. Valent B. (2015). How eukaryotic filamentous pathogens evade plant recognition. Curr. Opin. Microbiol. 26, 92–101. doi: 10.1016/j.mib.2015.06.012 26162502

[B4] Zhang S. Li C. Si J. Han Z. Chen D. (2022). Action mechanisms of effectors in plant-pathogen interaction. Int. J. Mol. Sci. 23, 6758. doi: 10.3390/ijms23126758 35743201 PMC9224169

